# *Purpureocillium jiangxiense* sp. nov.: Entomopathogenic Effects on *Ostrinia furnacalis* and *Galleria mellonella*

**DOI:** 10.3390/microorganisms12061041

**Published:** 2024-05-21

**Authors:** Wei Chen, Yanhong Tang, Tongyi Liu, Hongwang Hu, Cuiyi Ou, Qiongbo Hu, Qunfang Weng

**Affiliations:** National Key Laboratory of Green Pesticide, South China Agricultural University, Guangzhou 510642, China; cw@stu.scau.edu.cn (W.C.); tyh@stu.scau.edu.cn (Y.T.); lty@stu.scau.edu.cn (T.L.); hhw9908@stu.scau.edu.cn (H.H.); ocy@stu.scau.edu.cn (C.O.)

**Keywords:** *Purpureocillium*, new species, corn moth, greater wax moth, entomopathogen

## Abstract

The genus *Purpureocillium* is renowned for its role in biocontrol and biotechnological applications. The identification of new species within this genus is crucial for broadening our understanding of its ecological roles and potential utility in sustainable agriculture. This study aimed to characterize a new species of *Purpureocillium*, isolated from soil in eastern China, and to evaluate its bioactivity against *Ostrinia furnacalis* (corn moth) and *Galleria mellonella* (greater wax moth). We utilized morphological characterization; molecular phylogenetic analysis employing ITS, *nrLSU*, and *tef1* genes; and bioactivity assays to identify and characterize the new species. The newly identified species, *Purpureocillium jiangxiense* sp. nov., displays unique morphological and genetic profiles compared to known species. Bioactivity tests showed that this species exhibits inhibitory effects against *O. furnacalis* and *G. mellonella*, highlighting its potential in biocontrol applications. By the ninth day at a spore concentration of 1 × 10^8^ spores/mL, the mortality rate of the corn moth and greater wax moth reached 30% to 50% respectively. The discovery of *P. jiangxiense* sp. nov. adds to the genetic diversity known within this genus and offers a promising candidate for the development of natural biocontrol agents. It underscores the importance of continued biodiversity exploration and the potential for natural solutions in pest and disease management.

## 1. Introduction

Fungi serve crucial roles in nature and human life and have significant implications across various domains, including agriculture, medicine, industry, and food science [1]. Research on fungal diversity has advanced significantly over the past few decades; it continues to encounter numerous limitations and challenges [2]. Although more than 200,000 fungal species have been described, scientists believe that this figure represents only a fraction of the actual diversity. Suggesting that many species are yet to be discovered or described hinders our comprehensive understanding of global fungal diversity (https://gcm.wdcm.org/statistics, accessed on 7 January 2024).

The *Purpureocillium*, a genus in the Ascomycota phylum, Eurotiomycetes class, Hypocreales order, and Ophiocordycipitaceae family, was delineated from the species of *Paecilomyces lilacilus* based on its medical importance [3]. To date, this genus comprises six species (https://www.mycobank.org/, accessed on 7 May 2024), which are pivotal in environmental biology and applied microbiology, playing diverse and intricate roles within natural ecosystems and anthropogenic activities [4,5]. Notably, *P. lilacinum*, one of the most renowned species within this genus, is widely recognized for its potential applications in agricultural biotechnology and environmental science [6,7]. In the realm of biocontrol research, the *Purpureocillium* fungi have demonstrated remarkable capabilities in combating phytopathogenic nematodes, such as *Meloidogyne* spp. [8,9]. *Purpureocillium* species employ unique biological mechanisms, such as secreting enzymes capable of degrading nematode eggshells, effectively diminishing nematode populations and alleviating their damage to crops [10]. This natural and efficient control strategy offers a sustainable alternative to chemical pesticides, enhancing the sustainable development of agricultural ecosystems [11]. Additionally, *P. lilacinum* exhibits significant insecticidal activity and shows potential for effectiveness against a diverse array of pests. Studies demonstrated that this fungus has a significant insecticidal effect on insect and mite pests, such as *Myzus persicae* (Spinach aphid), *Spodoptera frugiperda* (Fall Armyworm), *Anastrepha ludens* (Mexican fruit fly), *Edessa rufomarginata* (Turquoise shield bug), the two-spotted spider mite *Tetranychus urticae*, etc. [12,13,14,15].

To exploit the resources of the *Purpureocillium* fungi, in this study, a new species was identified through classical morphological methods combined with molecular biology techniques. In addition, the insecticidal activity of this fungus against the corn moth, *Ostrinia furnacalis,* and greater wax moth, *Galleria mellonella*, was evaluated. This discovery significantly enhances the species diversity of the *Purpureocillium* genus and highlights its potential for biological control.

## 2. Materials and Methods

### 2.1. Soil Sample Collection

Soil samples were collected from the eastern region of China in June 2023, using a five-point sampling method to obtain 10–20 cm of surface soil at each site. Each sample, weighing approximately 100 g, was sealed in a self-sealing bag. The longitude, latitude, specific geographical location, and vegetation type of each collection point were recorded (Table 1). The samples were then stored at 4 °C.

### 2.2. Fungal Isolation and Culture

The collected soil samples were sieved through a 0.45 mm mesh to separate the fine soil. Two 10 g soil samples were each mixed to 100 mL of 0.1% Tween-80 solution. The mixture was vortexed to homogenize and then left to stand for 10 min to prepare the soil suspension. A total of 100 μL of the soil suspension was inoculated into selective media, evenly spread, and the process was repeated three times for each treatment. The samples were incubated in a constant temperature incubator at 26 ± 1 °C until single colonies appeared on the selective media (Potato 200 g, glucose 20 g, agar 20 g, chloramphenicol 50 mg, cicloheximide 50 mg, 3,4,5,6-Tetrachlorofluorescein 50 mg, and distilled water 1 L). Single colonies were then transferred to PDA medium (Potato 200 g, glucose 20 g, agar 20 g, and distilled water 1 L) using an inoculating loop to establish pure cultures. This procedure was repeated until the strains were purified. The purified *Purpureocillium* strains were inoculated into 20% glycerol solution and stored at −80 °C [16].

### 2.3. Morphological Observations

The purified strains were used to create 5 mm diameter fungal plugs and placed on various agar plates: PDA, Czapek’s (Sodium nitrate 3.0 g, dipotassium hydrogen phosphate 1.0 g, magnesium sulfate heptahydrate 0.5 g, potassium chloride 0.5 g, ferrous sulfate heptahydrate 0.01 g, sucrose 30.0 g, agar 15.0 g, and distilled water 1 L.), MEA (Malt extract powder 130.0 g, agar 15.0 g, chloramphenicol 0.1 g, and distilled water 1 L.), CYA (Sodium nitrate 3.0 g, dipotassium hydrogen phosphate 1.0 g, magnesium sulfate heptahydrate 0.5 g, yeast powder 5.0 g, potassium chloride 0.5 g, ferrous sulfate heptahydrate 0.01 g, sucrose 30.0 g, agar 15.0 g, and distilled water 1 L), and DG18 agar plates (Monopotassium phosphate 1.0 g, casein peptone 5.0 g, magnesium sulfate 0.5 g, anhydrous glucose 10.0 g, chloramphenicol 0.1 g, nitrofurazone 0.002 g, agar 15.0 g, and distilled water 1 L). These plates were incubated at 26 ± 1 °C for 7 days. The colony size and morphological characteristics were observed and recorded. The microstructural photography involved an optical microscope and scanning electron microscope (SEM). The sample preparation steps for the SEM are as follows: 5 mm diameter fungal plugs were inoculated onto PDA plates, with sterilized aluminum foil pieces around them, and then incubated in a dark incubator at 26 ± 1 °C until 2/3 of the aluminum foil was covered with mycelia. The aluminum foil pieces with the grown mycelia and spores were then fixed in an electron microscope fixative solution. After dehydration, drying, and sputtering with gold, the samples were photographed using a scanning electron microscope.

### 2.4. DNA Extraction, PCR, and Sequencing

The purified strains were inoculated onto PDA plates and incubated at 26 ± 1 °C for 7 days. After incubation, the fungal biomass was harvested from the plate surfaces and the genomic DNA was extracted using a combination of the CTAB method and silica column purification. The extraction should be performed according to the procedures outlined in the Omega HP Fungal DNA Kit D 3195-02 (Omega Bio-Tek, Guangzhou, China) instruction manual. Some modifications were made, and the specific steps are as follows: The scraped microbial cells were placed into a 1.5 mL centrifuge tube, 100 μL of CPL Buffer was immediately added, and a handheld tissue homogenizer was used to grind it for 30 s. The grinding time can be adjusted based on the extent of grinding. After sufficient homogenization, 500 μL of CPL Buffer and vortex were added to the mix and the sample was lysed. The tube was incubated in a 65 °C water bath for 30 min to assist lysis, inverting the tube twice during the period for thorough mixing. A total of 600 μL of chloroform:isoamyl alcohol (24:1) mixture was added, vortexed to mix, and centrifuged at 12,000 rpm for 10 min. A total of 300 μL of the supernatant was carefully transferred to a new 1.5 mL centrifuge tube, taking care to avoid disturbing the pellet. Then, 150 μL of CXD Buffer and 300 μL of anhydrous ethanol were added and vortexed to mix. A HiBind DNA column was placed in a 2 mL collection tube and the digest from step 5 was transferred into the column and centrifuged at 10,000 rpm for 1 min. The flow-through was discarded. The HiBind DNA column was returned to the 2 mL collection tube and 650 μL of SPW Wash Buffer was added and centrifuged at 10,000 rpm for 1 min. The flow-through was discarded. The steps described above were repeated. It was centrifuged at 14,000 rpm for 2 min with no buffer (dry spin). The column was placed in a 1.5 mL centrifuge tube and left to stand for 2 min. A total of 50 μL of preheated (65 °C) Elution Buffer was added to the center of the membrane and left to stand for 3 min and then centrifuged at 10,000 rpm for 1 min. The DNA concentration was measured and stored at −20 °C for later use.

The ribosomal internal transcribed spacer (ITS) region, the large ribosomal subunit (*nrLSU*), and Translation Elongation Factor 1-alpha (*tef1*) were amplified using the primers ITS1 (TCCGTAGGTGAACCTGCGG)/ITS4 (TCCTCCGCTTATTGATATGC) [17], LR0R (GTACCCGCTGAACTTAAGC)/LR5 (ATCCTGAGGGAAACTTC) [18], and EF983F (AGTTCGAGGCTGGTATCTCC)/EF2218R (CCTTGACGGAGACGTTCTT) [19], respectively.

The PCR reaction mixture (25 μL) consisted of 22 μL of 1.1 × S4 Fidelity PCR Mix (Genesand), with 1 μL each of forward and reverse primers and a DNA template. The PCR amplification conditions were as follows: an initial denaturation at 95 °C for 5 min, followed by 35 cycles of 95 °C for 30 s, annealing at 56 °C for 45 s, and extension at 72 °C for 1 min, with a final extension at 72 °C for 10 min. The PCR products were verified by 1% agarose gel electrophoresis, and those with clear single bands were sent to YouKang Biotech for Sanger sequencing. The sequenced gene sequences were uploaded to the GenBank nucleotide database of the NCBI, and the GenBank accession numbers were recorded.

### 2.5. Phylogenetic Analyses

A multigene phylogenetic analysis of *Purpureocillium* strains was conducted based on the ITS *nrLSU* and *tef1* sequences, using the sequences of related fungal-type species from the NCBI as the reference strains (Table 2). *Cordyceps gunnii* ARSEF 6828, also belonging to the Hypocreales, serve as an outgroup of phylogenetic trees. The sequence alignment was conducted online using MAFFT V 7 (https://mafft.cbrc.jp/alignment/server/ (accessed on 7 December 2023)) with the iterative refinement method (FFT-NS-i) for multiple sequence alignment (MSA). The alignments were checked and calibrated on MEGA V 7.0. Subsequently, phylogenetic analyses and tree construction were performed using PhyloSuite V 1.2.3 [20]. Both the maximum likelihood (ML) and Bayesian Inference (BI) methods were employed for the phylogenetic analyses. Initially, the aligned sequences were trimmed using Gblocks V 0.91b to remove poorly aligned positions [21]. Then, ModelFinder V 2.2.0 was used to select the optimal partition strategies and evolutionary models for both IQ-TREE and MrBayes, which were employed for phylogenetic tree construction [22]. The ML phylogenetic tree was constructed using IQ-TREE V 2.2.0, with the bootstrap analysis performed 1000 times to assess the stability of the tree topology [23]. For the BI phylogenetic tree construction, MrBayes V 3.2.7a was utilized. To ensure reliable results, the Markov Chain Monte Carlo (MCMC) was set to run for 2,000,000 generations, sampling every 1000 generations, with the first 25% of the runs discarded as burn-in [24]. The resulting phylogenetic trees from the ML and BI analyses were visualized using MEGA V 7.0 and Fig Tree V 1.4.3.

### 2.6. Virulence Assay of Purpureocillium Isolates

The test *G. mellonella* and *O. furnacalis* were reared through laboratory generations. The diet for *G. mellonella* consisted of 50 g of milk powder, 20 g of yeast powder, 100 g of flour, 100 g of wheat bran, 100 g of cornmeal, 50 g of beeswax, 50 g of honey, and 60 g of glycerin. The diet for the *O. furnacalis* included 43 g of cornmeal, 43 g of soybean flour, 26 g of yeast extract, 26 g of glucose, 1.5 g of a multivitamin complex, 5.7 g of agar, and 1.5 g of sorbic acid.

For the bioactivity testing of the *Purpureocillium* species, the procedure was adapted from the Chinese agricultural industry standard, incorporating some modifications [25]. The fungal strain was transferred to PDA medium and cultured for 7 to 14 days. Following adequate sporulation, conidia were scraped from the colony surfaces and used to prepare a spore suspension with 0.1% Tween-80 solution to a concentration of 1 × 10^8^, 1 × 10^7^, 1 × 10^6^, and 1 × 10^5^ spores/mL. Healthy third-instar larvae of *O. furnacalis* and *G. mellonella* were then placed into sterilized centrifuge tubes, the spore suspension was added, and the tubes were quickly capped and inverted 10 times before transferring the larvae to disposable plastic bowls and incubated at a constant temperature of 25 ± 1 °C. The treated larvae were fed with feed, with the feed changed every two days. Larval mortality was recorded, and deceased larvae were transferred to a petri dish. After fungal hyphae grew on the insect body, they were re-inoculated onto PDA plates to verify if the death was caused by the fungi, thus completing Koch’s postulates. Control larvae treated with 0.1% Tween-80 solution were maintained, with 10 larvae per treatment and three repetitions. For statistical analysis, the software Excel 2010 (Microsoft, Washington, DC, USA) and DPS 9.5 (Data Processing System, Zhejiang, China) were employed.

## 3. Results

### 3.1. Morphological Features

**Figure 1:**
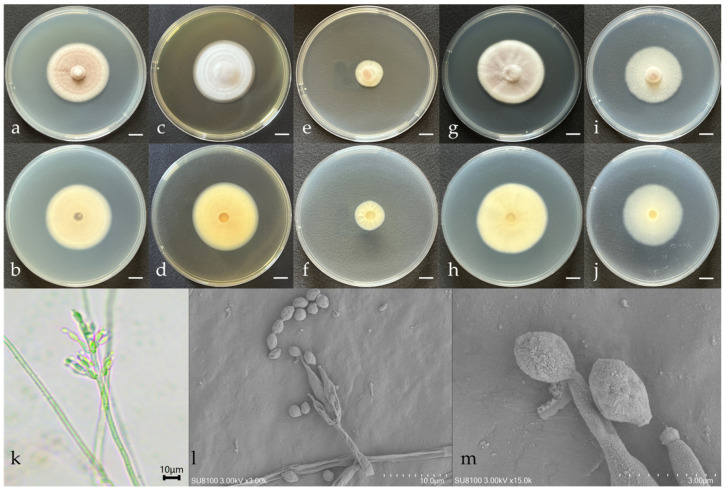
Morphological characteristics of *Purpureocillium jiangxiense* sp.nov. (**a**,**b**) Colony morphology after 7 days of cultivation on PDA; (**c**,**d**) colony morphology after 7 days of cultivation on MEA; (**e**,**f**) colony morphology after 7 days of cultivation on DG18; (**g**,**h**) colony morphology after 7 days of cultivation on CYA; (**i**,**j**) colony morphology after 7 days of cultivation on Czapek; (**k**,**l**) sporulating structures and conidia; and (**m**) conidia. Scale: (**a**–**j**) = 1 cm.

The morphological characteristics and microscopic structures of the new species cultured on various media are depicted in Figure 1. The description of the fungal morphology is as follows:

*Purpureocillium jiangxiense* W. Chen & Q. Weng, sp. nov. from Jiangxi (Figure 1).

Nomenclature: Named after its geographical location.

Type Strain Information: Strain number JX13B01 (GDMCC 3.1070), isolated from forest soil in Meiling Town, Xinjian District, Nanchang City, Jiangxi Province, China, collected by Wei Chen in June 2023.

After 7 days of cultivation at 26 ± 1 °C in darkness, the colony on the PDA agar measured 41.33 ± 0.94 mm, with the inner circle being lilac-purple and the outer circle off-white and velvety in texture and the reverse side off-white (Figure 1a,b). On Czapek’s medium, the colony measured 37.67 ± 0.47 mm, with a light yellow flocculent surface and an off-white reverse (Figure 1i,j). On the CYA medium, the colony measured 41.67 ± 1.70 mm, forming a fairly regular circle, with a light pink velvety surface displaying radial striations and a light-yellow reverse (Figure 1g,h). On the DG18 medium, the colony measured 18.67 ± 0.47 mm, growing slowly, with a pale yellow velvety surface turning light pink in sporulating areas and a pale yellow reverse with distinct radial fissures (Figure 1e,f). On the MEA medium, the colony measured 42 ± 0.82 mm, with a fairly regular circular shape and clear annular rings on the surface, and the reverse was yellow (Figure 1c,d).

No sexual structures were observed when cultivated on the PDA, Czapek, CYA, DG18, and MEA media. The vegetative hyphae were hyaline, 3.02–3.77 μm wide. Conidiophores branched once forming short branches, which were whorled, hyaline, aseptate, and clavate with acute ends, 12.41–15.07 μm long and 0.88–3.10 μm wide; the short branches were terminated in single or double phialides, producing conidia exogenously, which were fusiform to nearly spherical, measuring 2.47–4.46 × 2.04–2.57 μm (Figure 1k–m).

### 3.2. Phylogenetic Analyses

Two strains of *Purpureocillium* sp. fungi were isolated from soil samples in Jiangxi provinces. The phylogenetic analysis included 10 strains of five *Purpureocillium* species downloaded from the NCBI and the 2 strains screened in this study. *C. gunnii* ARSEF 6828 was used as the outgroup. The final dataset consisted of sequence data totaling 1911 base pairs, comprising the ITS region (513 bp), the *nrLSU* region (831 bp), and the *tef1* region (567 bp).

**Figure 2:**
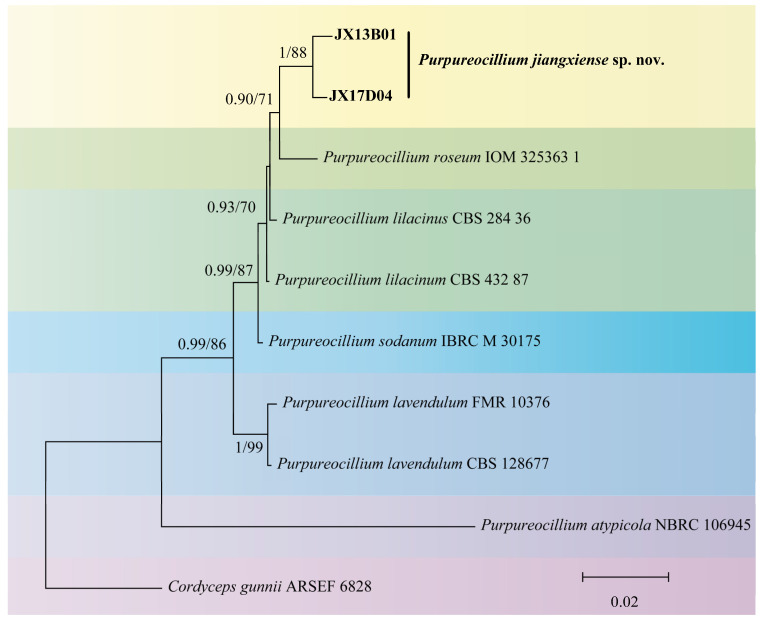
Phylogenetic tree of *Purpureocillium* based on multiple gene loci (ITS, *nrLSU*, and *tef1*) combined with maximum likelihood method.

For the Bayesian analysis, ModelFinder recommended using the HKY + F + G4 model, while for the IQtree analysis, ModelFinder recommended using the TN + F + G4 model. In the phylogenetic analysis based on three concatenated gene loci, the tested strains failed to cluster with any previously reported *Purpureocillium* species but formed a separate evolutionary branch. Therefore, these two strains are identified as one new species (Figure 2).

The phylogenetic analysis results indicate that *P. jiangxiense* and *P. roseum* are closely related, but they can be distinguished by morphological characteristics, such as spore size. The spores of *P. jiangxiense* are fusiform to nearly spherical (average 3.38 × 2.33 μm), which are larger than the spherical spores of *P. roseum* (average 2.25 μm). In addition, there are nucleotide differences between *P. jiangxiense* and *P. roseum* in ITS (12 bp) and *nrLSU* (6 bp).

### 3.3. Biological Activity of New Species of Purpureocillium

**Figure 3:**
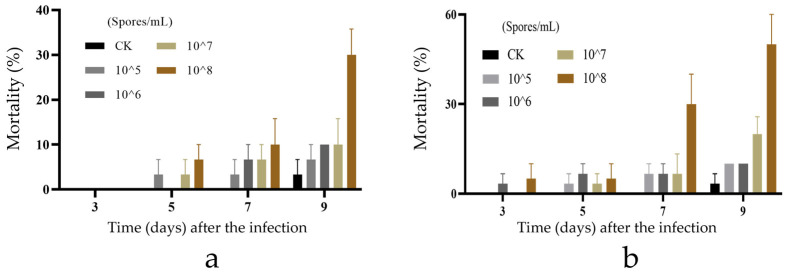
Biological activity of *P. jiangxiense* JX13B01 against the third-instar larvae of *O. furnacalis* at different concentrations. (**a**) Bioactivity test results for third instar *O. furnacalis* larvae, CK, 0.1% Tween-80 solution-treated larvae; (**b**) bioactivity test results for third instar *G. mellonella* larvae, CK, 0.1% Tween-80 solution-treated larvae.

The bioactivity test results of a new species of *Purpureocillium* against the third-instar larvae of *O. furnacalis* (Figure 3a) and *G. mellonella* (Figure 3b) are depicted in Figure 3. The insecticidal effect increased with the concentration of fungal spores. At a spore concentration of 10^8^ spores/mL, the strain demonstrated varying levels of activity against the two pests. Notably, *P. jiangxiense* exhibited enhanced bioactivity against the *G. mellonella*, achieving a mortality rate of 50% in the third-instar larvae by the ninth day.

**Figure 4:**
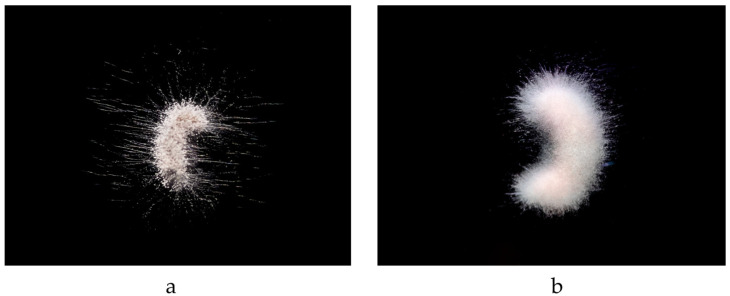
Morphological characteristics of fungal infection in insect. (**a**) Day four post-infection mortality in *O. furnacalis* larvae; (**b**) day four post-infection mortality in *G. mellonella* larvae.

The characteristics of the strain infecting two different insects are shown in Figure 4. Under the optical microscope, the infection of insects by *P. jiangxiense* is initially observed as white mycelium emerging from the dense, bristly areas of the insect’s thorax, thoracic legs, and intersegmental membranes. With time, the entire insect body becomes enveloped in white mycelium, which subsequently leads to the production of pale pink conidia.

## 4. Discussion

*Purpureocillium* is a newly established genus that was separated from *Paecilomyces*. Many strains of *P. lilacinum* are used as biological control agents against plant-pathogenic nematodes [26]. Additionally, some strains exhibit insecticidal activities, while others pose an infection risk to immunocompromised individuals [27]. Preliminary research suggests that *P. lilacinum* is the dominant species in nearly all habitats and regions, likely due to its ability to adapt to various environmental conditions, including varying temperatures, humidity levels, and soil pH values. This adaptability enables it to dominate across a wide range of geographic areas and ecosystems [28]. Furthermore, *P. lilacinum* is a well-recognized biocontrol agent, particularly effective against plant-pathogenic nematodes, which increases the prevalence of this fungal genus in the soil [29]. In contrast, strains of *P. lavendulum*, another member of the *Purpureocillium* genus, are relatively less common. This rarity is likely due to the thermal adaptability of *P. lavendulum*, which cannot withstand higher temperatures, thereby limiting its natural distribution [30].

Traditional morphological identification of fungi typically involves both macroscopic and microscopic observations of fungal spores, mycelia, and colonies, forming the basis of fungal identification [31]. However, this method is significantly influenced by environmental factors, such as changes in temperature and humidity, which can alter fungal morphological characteristics and complicate identification. Additionally, accurately identifying species with similar morphological features based solely on morphology can be challenging [32,33,34].

DNA barcoding technology was initially proposed in 2003 by Canadian researcher Paul Hebert and his team, who recommended using a standard DNA fragment as a “barcode” for species identification [35]. While this concept was originally developed for animals, it has been rapidly adopted for other biological groups, including fungi. However, single-gene analysis often encounters limitations due to the uniform evolutionary rate of that gene, which may not sufficiently distinguish subtle differences between closely related species or within long-evolved species groups [36,37]. In this study, we integrated preliminary morphological studies with ITS sequencing and found that the species of some strains could not be definitively identified.

As gene sequencing and analytical technologies advance, biologists are increasingly exploring phylogenetic analysis methods that utilize multiple genes, based on the assumption that different genes experience varied selective pressures during evolution. Such an integration of gene data can yield more accurate and stable phylogenetic relationships [38]. Early multigene analysis methods included concatenating multiple gene sequences into a “super gene” for analysis or integrating separate analyses for a collective judgment [39]. With the advent of the 21st century, with the advancement of computational biology and statistical methods, multigene analysis techniques have been further refined. These advances have led to model-based methods that consider the evolutionary signals of multiple genes simultaneously, rather than merely concatenating different gene sequences end-to-end [40]. In recent years, multigene joint analysis methods have been applied in the delineation of multiple fungal species and have successfully resolved various fungal communities. With the advancement of high-throughput sequencing technologies, it has become easier and more economical to obtain large amounts of genomic data, providing a rich resource for multigene phylogenetic tree construction and enabling the development of phylogenetic trees based on genomic data. This trend is likely to continue in the future for fungal species identification and delineation [41,42]. In this study, we extended previous research by integrating a multigene joint analysis on two *Purpureocillium* strains that could not be identified by a single gene locus alone. By combining this with a comprehensive assessment of classic morphological features, we successfully identified a previously unreported new species.

This study also conducted a preliminary assessment of the bioactivity of the newly identified species, demonstrating that the strain has significant biological activities against both *G. mellonella* and *O. furnacalis*. These results indicate the potential of the strain as a biocontrol agent. To fully understand the strain’s range of insecticidal activities, it is crucial to expand bioactivity tests to a wider array of pests. This expansion would entail evaluating the strain’s effectiveness against various insect orders and families to determine its range and specificity. Furthermore, exploring the mode of action through which the strain exerts its bioactivity—be it pathogenicity, toxin production, or other mechanisms—would provide valuable insights.

Further research should also include the optimization of cultivation conditions that maximize the production of bioactive compounds by the strain. Moreover, developing effective bioassay formulations and conducting field trials are crucial for maximizing the potential of newly discovered entomopathogenic fungi. Optimizing formulations can enhance the stability and adaptability of the product, increasing acceptance among farmers. Field trials effectively evaluate the effectiveness, environmental safety, and economic viability of the biopesticide under natural conditions, ensuring its success in commercialization. This further provides substantial support for the development of sustainable agriculture.

Ultimately, detailed bioactivity profiling will facilitate the development of targeted biopesticide formulations. This approach will not only enhance the efficacy of pest management strategies but also reduce the reliance on chemical pesticides, thus supporting sustainable agricultural practices. Comprehensive data will provide a robust foundation for advancing research and development in biocontrol agents.

## 5. Conclusions

This study successfully identified a new species within the genus *Purpureocillium*, *P. jiangxiense* sp. nov., using a combination of traditional morphological methods and molecular phylogenetic analysis. Furthermore, a preliminary evaluation of the insecticidal activity of this new species revealed its potential biological efficacy against specific pests, thereby demonstrating its value as a biocontrol agent. These findings not only enrich the fungal database in China but also offer important scientific evidence supporting the development of novel biocontrol agents in the future.

## Figures and Tables

**Table 1:** Sample information.

Sample Number	Strain	Collection Site	Vegetation	Longitude and Latitude
JX13B	JX13B01	Nanchang City, Jiangxi Province, China	forest	115°45′18″ E, 28°47′28″ N
JX17D	JX17D04	Fuzhou City, Jiangxi Province, China	orchard	116°59′11″ E, 27°21′32″ N

**Table 2:** Strain information used for phylogenetic analysis of fungi in the genus *Purpureocillium*.

Species	Voucher Information	GenBank Accession Number		
		ITS	*nrLSU*	*tef1*
*P. atypicola*	NBRC 106945	LC008212		LC008347
*P. lavendulum*	CBS 128677	MH864976	NG067468	
*P. lavendulum*	FMR 10376	FR734106	FR775489	FR775516
*P. lilacinum*	CBS 284.36	AY624189	MH876593	EF468792
	CBS 432.87	HQ842819	MH873778	AY624228
*P. roseum*	IOM 325363	MT560195	MT560197	
*P. sodanum*	IBRC-M 30175	KX668542		
*P. jiangxiense* sp.nov.	JX17D04	PP555636	PP555645	PP658209
*P. jiangxiense* sp.nov.	JX13B01T	PP555637	PP555646	PP658210
*C. gunnii*	ARSEF 6828	HM140630	HM140633	HM140636

## Data Availability

All relevant data are within the manuscript.
