# Correction: Chen et al. *Purpureocillium jiangxiense* sp. nov.: Entomopathogenic Effects on *Ostrinia furnacalis* and *Galleria mellonella*. *Microorganisms* 2024, *12*, 1041

**DOI:** 10.3390/microorganisms13122872

**Published:** 2025-12-18

**Authors:** Wei Chen, Yanhong Tang, Tongyi Liu, Hongwang Hu, Cuiyi Ou, Qiongbo Hu, Qunfang Weng

**Affiliations:** National Key Laboratory of Green Pesticide, South China Agricultural University, Guangzhou 510642, China; cw@stu.scau.edu.cn (W.C.); tyh@stu.scau.edu.cn (Y.T.); lty@stu.scau.edu.cn (T.L.); hhw9908@stu.scau.edu.cn (H.H.); ocy@stu.scau.edu.cn (C.O.)

There was an error in the original publication [1].

A correction has been made to 3. Results, 3.1. Morphological Features, Paragraph 2, the correct content appears below:

*Purpureocillium jiangxiense* W. Chen & Q. Weng, sp. nov., mycobank number, MB 664600 (Figure 1).

Another correction has been made to 3. Results, 3.1. Morphological Features, Paragraph 4, the correct content appears below:

Type Strain Information: Strain number JX13B01 (Holotype: GDMCC 3.1070 preserved in Guangdong Microbial Culture Collection Center with a metabolically inactive state) was isolated from forest soil in Meiling Town, Xinjian District, Nanchang City, Jiangxi Province, China, collected by Wei Chen in June 2023.

The authors state that the scientific conclusions are unaffected. This correction was approved by the Academic Editor. The original publication has also been updated.
